# Level, Uphill, and Downhill Running Economy Values Are Correlated Except on Steep Slopes

**DOI:** 10.3389/fphys.2021.697315

**Published:** 2021-07-01

**Authors:** Marcel Lemire, Mathieu Falbriard, Kamiar Aminian, Grégoire P. Millet, Frédéric Meyer

**Affiliations:** ^1^Faculty of Medicine, Translational Medicine Federation, University of Strasbourg, Strasbourg, France; ^2^Faculty of Sport Sciences, University of Strasbourg, Strasbourg, France; ^3^Institut de Recherche en Informatique, Mathématiques, Automatique et Signal (IRIMAS), University of Haute-Alsace, Mulhouse, France; ^4^Laboratory of Movement Analysis and Measurement, Swiss Federal School of Technology (EPFL), Lausanne, Switzerland; ^5^Institute of Sport Sciences, University of Lausanne, Lausanne, Switzerland; ^6^Digital Signal Processing Group, Department of Informatics, University of Oslo, Oslo, Norway

**Keywords:** energy cost, biomechanics, running gait, muscle strength, ground reaction forces, treadmill

## Abstract

The aim of this study was first to determine if level, uphill, and downhill energy cost of running (ECR) values were correlated at different slopes and for different running speeds, and second, to determine the influence of lower limb strength on ECR. Twenty-nine healthy subjects completed a randomized series of 4-min running bouts on an instrumented treadmill to determine their cardiorespiratory and mechanical (i.e., ground reaction forces) responses at different constant speeds (8, 10, 12, and 14 km·h^−1^) and different slopes (−20, −10, −5, 0, +5, +10, +15, and +20%). The subjects also performed a knee extensor (KE) strength assessment. Oxygen and energy costs of running values were correlated between all slopes by pooling all running speeds (all *r*^2^ ≥ 0.27; *p* ≤ 0.021), except between the steepest uphill vs. level and the steepest downhill slope (i.e., +20% vs. 0% and −20% slopes; both *p* ≥ 0.214). When pooled across all running speeds, the ECR was inversely correlated with KE isometric maximal torque for the level and downhill running conditions (all *r*^2^ ≥ 0.24; *p* ≤ 0.049) except for the steepest downhill slope (−20%), but not for any uphill slopes. The optimal downhill grade (i.e., lowest oxygen cost) varied between running speeds and ranged from −14% and −20% (all *p* < 0.001). The present results suggest that compared to level and shallow slopes, on steep slopes ~±20%, running energetics are determined by different factors (i.e., reduced bouncing mechanism, greater muscle strength for negative slopes, and cardiopulmonary fitness for positive slopes). On shallow negative slopes and during level running, ECR is related to KE strength.

## Introduction

Running economy is considered as a key factor of road running performance such as marathon (Saunders et al., 2004; Joyner and Coyle, 2008; Jones et al., 2020). It is generally described by values of energy cost of running (ECR, i.e., the metabolic energy spent per unit of distance covered, expressed in J·kg^−1^·m^−1^) (Barnes and Kilding, 2015), since it allows taking into consideration not only oxygen consumption ($\dot{\text{V}}{\text{O}}_{2}$) but also substrate oxidation (Fletcher et al., 2009).

Nevertheless, contrary to running track and road races, the importance of running economy in ultra-trail running competitions remains debated (Millet, 2012; Millet et al., 2012). Moreover, trail running performance remains difficult to predict due to the variety of distances, conditions, terrains, physiological and biomechanical factors (such as ECR, gait spatiotemporal parameters, and muscular torque). Thus, additional data on the contribution of both metabolic and mechanical aspects of ECR in conditions relevant to trail running (e.g., at various slopes, both uphill and downhill, and at various speeds) are required.

Level and uphill oxygen cost of running (OCR, i.e., the oxygen consumption per unit of distance covered, expressed in mLO_2_·kg^−1^·km^−1^) were shown to be positively correlated in elite trail runners (Willis et al., 2019), but not in their sub-elite counterparts (Balducci et al., 2016, 2017), and this point remains unclear in healthy people unaccustomed to inclined running. Moreover, whether downhill ECR is related to the level and uphill ECR remains an open question due to the scarcity of results on this topic. Only Breiner et al. (2018) reported a relationship between level, uphill, and downhill OCR (at 0, +7.5, and −5% slopes, respectively). They reported that strong correlations between slopes likely arise from the homogeneity in the subjects, who were athletes accustomed to hill running with a similar training/practice exposure to level, uphill, and downhill terrain, and equal skill and/or physiological adaptations.

During downhill running, Minetti et al. (1994) have highlighted that OCR values decreased and attained an optimum value at a −20% slope and then increased again on steeper negative slopes. Two mechanisms explained this observation. First, the vertical ground reaction forces and the ratio between muscle positive and negative work (i.e., concentric vs. eccentric muscle actions) increase or decrease proportionally to the slope of the terrain (Minetti et al., 1994; Dewolf et al., 2016). Second, at both high negative and positive slopes, the bouncing mechanism is reduced, and the efficiency values then reflect just the muscle positive work in uphill as well as the negative work in downhill (Minetti et al., 1994; Dewolf et al., 2016), resulting in the deterioration of the OCR on steep negative slopes.

Moreover, it has been demonstrated that lower limb strength, particularly of the knee extensor (KE) muscles, is widely involved in downhill running and is correlated with downhill running time-trial performance (Lemire et al., 2021). Maximal strength is known to influence ECR in level running and triathlon (Millet et al., 2002). Logically, downhill ECR is also likely influenced by ECR strength. Nevertheless, it remains unclear whether lower limb strength is correlated with ECR at the different running slopes.

Therefore, the aims of this study were first to determine if level, uphill, and downhill ECR values were correlated at different slopes and for different speeds, and second, to determine the influence of lower limb strength on ECR values. We tested two hypotheses: (i) ECR values would be correlated except on the steepest slopes, where mechanical constraints are more specific, and (ii) downhill ECR would be related to KE strength.

## Materials and Methods

### Subjects

A group of 29 healthy people (19 males and 10 females) volunteered in this study [age: 34 ± 10 (mean ± SD) years; height: 1.74 ± 0.09 m; body mass: 68.3 ± 12.2 kg; maximal oxygen uptake ($\dot{\text{V}}{\text{O}}_{2\text{max}}$): 56.6 ± 8.9 ml·min^−1^·kg^−1^; velocity at $\dot{\text{V}}{\text{O}}_{2\text{max}}$: 16.7 ± 2.7 km·h^−1^]. Participants represented a wide range of aerobic fitness, running between one and five times a week. They were all familiar with treadmill running but were not trail specialists. All participants were informed of the benefits and risks of this investigation before giving their written informed consent to participate in this study. The experiment was previously approved by our Institutional Review Board (CCER-VD 2015-00006) and complied with the Declaration of Helsinki.

### Experimental Design

Each subject visited the laboratory for four experimental sessions: (i) a level running (0% slope), incremental test, and a KE strength assessment; (ii) three sessions with seven to eight running bouts of 4 min each, at constant speeds (8, 10, 12, or 14 km·h^−1^) and slopes (−20, −10, −5, 0, +5, +10, +15, or +20%) in a randomized order, corresponding to 25 different conditions, since the extreme-intensity conditions (i.e., ≥10 km·h^−1^ at +20%, ≥ 12 km·h^−1^ at +15%, ≥ 14 km·h^−1^ at +10% slope) and a walking condition (i.e., 8 km·h^−1^ at −20% slope) were excluded. The −15% slope was not tested in order to limit the number of downhill conditions and the subsequent eccentric-induced muscle damages that may alter ECR. The sequence of trials within each session was randomized for each participant. All sessions were performed at the same time of the day and separated by 1 week of recovery. The subjects were instructed to not perform any eccentric and/or strenuous exercises in this time interval.

### Maximal Incremental Level Running Test and Constant Velocity Running Bouts in Level, Uphill, and Downhill

All running sessions were performed on an instrumented treadmill (T-170-FMT, Arsalis, Belgium). At the first session, all participants performed an incremental running test until exhaustion. The first stage began at 8 km·h^−1^ for 4 min and then increased by 1 km·h^−1^ every min. For each session, oxygen uptake ($\dot{\text{V}}{\text{O}}_{2}$), carbon dioxide output, ventilation, respiratory frequency, tidal volume, and respiratory exchange ratio were collected breath-by-breath through a facemask with an open-circuit metabolic cart with rapid O_2_ and CO_2_ analyzers (Quark CPET, Cosmed, Rome, Italy). $\dot{\text{V}}{\text{O}}_{2\text{max}}$ was defined as the highest 30 s $\dot{\text{V}}{\text{O}}_{2}$ value during the maximal incremental test. The speed associated with $\dot{\text{V}}{\text{O}}_{2\text{max}}$ was determined as the minimal speed associated with $\dot{\text{V}}{\text{O}}_{2\text{max}}$ (Billat and Koralsztein, 1996). About 30 s after the end of the test, the rated perceived exertion scale was used to assess the intensity of the test. Before each session, the pneumotachograph and the O_2_ and CO_2_ analyzers were calibrated according to the manufacturer's instructions. Heart rate was continuously measured (Polar Electro, Kempele, Finland).

### Energy Cost of Running Trials

As indicators of running economy, net OCR and net ECR were established for each running condition. For conditions where the intensity was higher than 1.00 for respiratory exchange ratio or blood lactate higher than 4.0 mmol·L^−1^, a $\dot{\text{V}}{\text{O}}_{2}$ correction has been applied (di Prampero, 1981), even though it is still debated (Poole et al., 2021). Resting $\dot{\text{V}}{\text{O}}_{2}$ ($\dot{\text{V}}{\text{O}}_{2\text{rest}}$) was averaged over the final minute of a 3-min baseline during which the subjects were standing quietly on the treadmill, before the start of the incremental test. Average values of net OCR and ECR were calculated between 3:15 and 3:45 (min:s) of each bout as follows (Fletcher et al., 2009):

(1) $$\begin{array}{cc} Net\;OCR & =\frac{\dot{\text{V}}{\text{O}}_{2\;steady-state}-\dot{\text{V}}\text{O}2\;rest}{\text{v}}\;\times 60 \end{array}$$

(2) $$\begin{array}{cc} Net\;ECR & =\frac{\dot{\text{V}}{\text{O}}_{2\;steady-state}-\dot{\text{V}}\text{O}2\;rest}{\text{v }*\;1000}\;\times 60\;\times \text{E}\left(O_{2}\right) \end{array}$$

where *Net OCR* is expressed in mlO_2_·kg^−1^·km^−1^, *Net ECR* in J·kg^−1^·m^−1^, $\dot{\text{V}}$O_2steady−state_ for the oxygen consumption at steady state, and $\dot{\text{V}}$O_2rest_ for the oxygen consumption at baseline in mlO_2_·kg^−1^·min^−1^, v for treadmill speed in km·h^−1^ and E(O_2_) for the energy equivalent of O_2_ estimated by the respiratory exchange ratio (Minetti et al., 2002).

The breathing duty cycle is the ratio between the inspiration time and the total cycle ventilation time. The optimum treadmill slope, as the lowest OCR, was calculated for each speed as the lowest solution of Minetti et al. (1994):

(3) $$\begin{array}{c} Optimum\;slope\;=\;\frac{-2c\;\pm \sqrt{4c^{2}-12bd}}{6d} \end{array}$$

where b, c, and d belong to the equation *OCR* = a + b*i* + c*i*^2^ + d*i*^3^, corresponding to a third-order polynomial function (*i* is the slope in percent).

### Blood Lactate Analyses

Blood lactate concentration was assessed from finger blood samples (Lactate Scout+, EKF Diagnostics, Leipzig, Germany) before the maximal incremental running test and after 1 and 3 min of recovery. For the constant-speed running bouts sessions, the blood samples were collected after 3 min of recovery for conditions with energy cost over the first ventilatory threshold. When appropriate, blood lactate values were used for $\dot{\text{V}}{\text{O}}_{2}$ correction (di Prampero, 1981).

### Knee Extensor Muscles' Torque Assessment

Before the maximal incremental running test and after 1 and 5 min of recovery, as well as before and 5 min after each of the three last sessions, each subject performed the isometric maximal voluntary contractions with the major extensor muscle groups of the lower limb on a custom-built chair ergometer equipped with a force gauge (Universal Load Cell, VPG Revere transducers, Germany) at the ankle. Participants sat with a 90° hip angle with the right knee positioned at 90° of flexion (0° = fully extended). The lever arm was attached 2 cm above the malleolus with a stiff strap. To prevent the upper body movement, participants crossed their arms across their chest and were stabilized with a stiff strap that wrapped around their trunk. The force obtained from strain-gauge transducer was recorded (MP150, Biopac System, Santa Barbara, CA) with an acquisition frequency of 1,000 Hz. The corresponding torque was then calculated and stored for analysis with dedicated software (AcqKnowledge 4.2 for MP systems, Biopac System, Santa Barbara, CA). To ensure the reliability of the measurements across sessions, the ergometer's participant settings were kept constant between sessions. Before the maximal voluntary contraction performed at the beginning of each session, the participant performed several voluntary contractions for warmup. Then, two 5-s maximal efforts, with 1 min rest in between, were performed while being verbally encouraged. A third maximal effort was performed if the second was better than the first one, and the highest score reached was retained. For the maximum voluntary contractions performed during the recovery, only one repetition was asked. All measurements were performed by the same two experienced investigators.

### Biomechanics Data Collection and Processing

An instrumented treadmill (T-170-FMT, Arsalis, Belgium) equipped with a three-dimensional force platform and sampling at 1,000 Hz was used in this study to obtain the maximum vertical to the earth ground reaction force (F_z_) and vertical displacement of the center of mass during ground contact (Δy). To reduce the noise inherent to the treadmill's vibrations, we first applied a second-order band-pass Butterworth filter (25–65 Hz) (Falbriard et al., 2018) to the vertical ground reaction force signal. All data analyses were conducted by using MATLAB software version R2019a (MathWorks Inc., Natick, MA, United States).

The instants of initial contact and terminal contact were identified using a threshold of 7% of bodyweight on the filtered vertical ground reaction force signal (i.e., ~50 N), based on a previously published work (Falbriard et al., 2018). Initial and terminal contacts of the left and right legs were combined to determine different spatiotemporal parameters. The contact time (in milliseconds) is the time between the initial and terminal contacts of the same leg; the flight time (in milliseconds) is the time between the terminal contact of one leg and the initial contact of the other leg. The step frequency (in Hz) is the reciprocal of the time required for one step (time between two consecutive initial contacts). Finally, the step length (cm) is the quotient of the treadmill belt speed divided by the step frequency. The biomechanical duty cycle was calculated by dividing contact time by stride time. These data were continuously saved for 30 s between 3:15 and 3:45 (min:s) of the trial and averaged for each condition.

### Statistical Analysis

Linear mixed models were used to determine if there were differences in the dependent variables in between treadmill slopes and running speeds as fixed effects across trials (Jamovi 1.2, Sydney; Australia), with a subject identifier used as the random grouping effect to account for repeated measures on the same individuals. All variables were first examined for normality using a Shapiro–Wilk test and then standardized as Z-scores. Bonferroni's *post-hoc* test was used to compare within condition values. Pearson's product–moment correlation coefficients (*r*) and thresholds of 0.1, 0.3, and 0.5 for small, moderate, and large *r* (Cohen, 1988) were used to assess the intensity of the relations between variables using Statistica (13.5, Tulsa, Oklahoma, United States). For all these analyses, *p* < 0.05 was considered statistically significant, and *p* < 0.1 was considered as a tendency. All data are expressed as mean ± SD.

## Results

### Oxygen and Energy Costs in Inclined Running

All cardiorespiratory and biomechanical parameters were pooled across running speeds and are summarized in Table 1.

**Table 1:** Cardiorespiratory and biomechanical parameters by pooling all running speeds.

**Treadmill slope (%)**	**−20**	**−10**	**−5**	**0**	**+5**	**+10**	**+15**	**+20**
N	21	29	26	29	26	29	16	6
Corrected $\dot{\text{V}}{\text{O}}_{2}$ (ml·min^−1^·kg^−1^)	26.5 ± 4.4^#^	28.8 ± 3.5^#^	32.5 ± 3.0^*^	39.9 ± 3.5^*^	47.1 ± 4.4^*^	53.6 ± 4.0^#^^$^	58.2 ± 9.3^#^^$^	67.5 ± 4.2^#^
Respiratory exchange ratio (%)	0.77 ± 0.05^#^	0.78 ± 0.03^#^	0.81 ± 0.04^*^	0.86 ± 0.05^*^	0.90 ± 0.06^*^	0.94 ± 0.03^#^^$^	0.95 ± 0.06^#^^$^	1.00 ± 0.03^#^^$^
V_E_ (l·*min*^−1^)	56.0 ± 9.3^#^	54.8 ± 9.4^#^	60.2 ± 10.1^#^	73.1 ± 13.3^*^	86.5 ± 16.7^*^	100.1 ± 17.9^#^^$^	107.4 ± 20.7^#^^$^	120.4 ± 18.9^#^^$^
RF (breaths·*min*^−1^)	46 ± 10^#^	39 ± 7^#^^$^	37 ± 7^$^	36 ± 8^$^	39 ± 8^$^	41 ± 9^#^^$^	39 ± 7^$^	40 ± 7
HR (bpm)	133 ± 17^#^	129 ± 20^#^	132 ± 16^#^	144 ± 19^$^	156 ± 14^#^^$^	167 ± 15^#^^$^	169 ± 12^#^^$^	174 ± 10^#^^$^
b[La] (mmol·*l*^−1^)	−	−	3.0 ± 1.5	3.5 ± 1.2	4.3 ± 2.4	5.2 ± 1.6^#^	5.6 ± 3.0^#^	7.5 ± 1.9^#^
RPE	10 ± 2	9 ± 1	9 ± 2^#^	11 ± 2^*^	12 ± 2^$^	14 ± 1^$^	15 ± 2^$^	16 ± 2^$^
Step length (cm)	116 ± 10	112 ± 7	113 ± 6	112 ± 6	104 ± 11^$^	93 ± 8^#^^$^	86 ± 9^#^^$^	82 ± 3^#^^$^
Step frequency (Hz)	2.76 ± 0.10	2.70 ± 0.11^#^^$^	2.72 ± 0.12	2.76 ± 0.11	2.76 ± 0.14	2.79 ± 0.13	2.79 ± 0.13	2.71 ± 0.09
Contact time (ms)	255 ± 29^#^	273 ± 25	275 ± 24^$^	274 ± 25^$^	285 ± 27^$^	298 ± 29^#^^$^	301 ± 25^#^^$^	322 ± 12^#^^$^
Aerial time (ms)	107 ± 32^#^	98 ± 21^#^	95 ± 18	89 ± 20^$^	78 ± 17^$^	62 ± 20^#^^$^	58 ± 22^#^^$^	48 ± 12^#^^$^
Fz (N)	1,467 ± 225	1,438 ± 232	1,430 ± 243	1,409 ± 252	1,347 ± 240^$^	1,259 ± 193^#^^$^	1,206 ± 193^#^^$^	1,194 ± 106^#^^$^
Δy (cm)	6.3 ± 0.6	6.6 ± 0.5^#^^$^	6.5 ± 0.5^#^	6.2 ± 0.5	6.1 ± 0.6	5.8 ± 0.5^#^^$^	5.7 ± 0.7^#^^$^	5.9 ± 0.6

*Values are means ± SD, corrected oxygen uptake (corrected $\dot{\text{V}}{\text{O}}_{2}$) from baseline oxygen uptake ($\dot{\text{V}}{\text{O}}_{2\text{rest}}$) 5.0 ± 0.6 ml·min^−1^·kg^−1^, minute pulmonary ventilation (V_E_), respiratory frequency (RF), heart rate (HR), blood lactate concentration (b[La]), rate of perceived exertion (RPE), maximal vertical ground reaction force (Fz), vertical displacement of the center of mass during ground contact (Δy). Asterisks (*

* *) indicate a statistically significant difference vs. all other conditions*,

# *vs. 0% slope, and*

$ *vs. −20% slope (p < 0.05)*.

**Figure 1:**
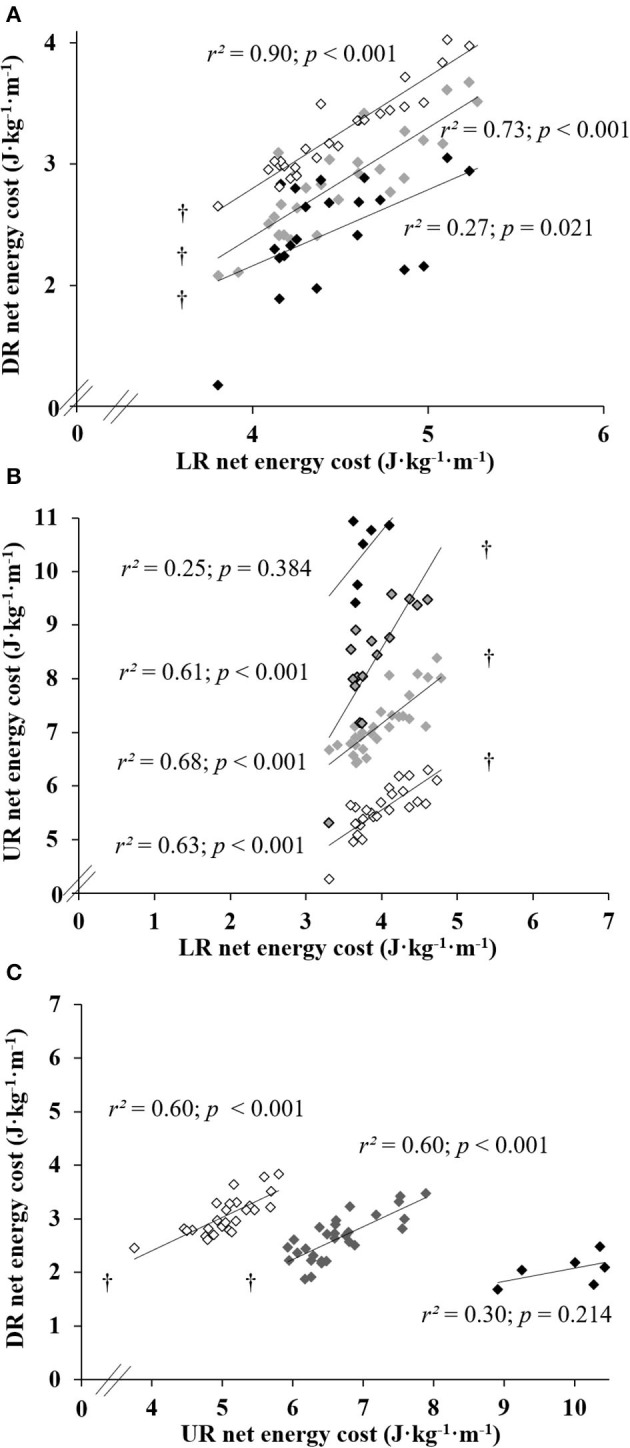
Energy cost of running relationships between level running (LR) and downhill (DR) **(A)**, between LR and uphill running (UR) **(B)** and between UR and DR **(C)**. Each point represents a speed averaged ECR of a given subject who sustained the exercise at same (absolute) slope value ↓±5%, υ±10%, υ+15% and υ±20%; † indicate a statistically significant correlation (*p* < 0.05).

When pooled across all running speeds, ECR values were largely correlated between level, uphill, and downhill at all slopes (Figure 1; all *p* ≤ 0.021), except on +20% slope compared to level and downhill at the same slope (Figure 1; both *p* ≥ 0.214).

For a given running speed (i.e., 8, 10, 12, or 14 km·h^−1^), ECR values correlated moderately to strongly between level, uphill, and downhill (all *r*^2^ ≥ 0.23; *p* ≤ 0.049), except at several steep slopes compared to level condition (i.e., −20% slope at 10 and 14 km·h^−1^, +10% slope at 12 km·h^−1^, and +20% slope at 8 km·h^−1^, all *p* ≥ 0.055).

Due to the difficulty of the task, the number of subjects who were able to perform the exercise bout at the steepest positive slope (i.e., +20%) was logically reduced (*N* = 6 vs. up to *N* = 29 in the other conditions). The average maximal oxygen uptake ($\dot{\text{V}}{\text{O}}_{2\text{max}}$) for the 29 subjects was 56.6 ± 8.9 ml·min^−1^·kg^−1^, and their velocity associated with $\dot{\text{V}}{\text{O}}_{2\text{max}}$ was 16.7 ± 2.7 km·h^−1^. The average $\dot{\text{V}}{\text{O}}_{2\text{max}}$ for the six subjects who performed the running bout on the +20% slope was 65.9 ± 6.6 ml·min^−1^·kg^−1^, and their velocity associated with $\dot{\text{V}}{\text{O}}_{2\text{max}}$ was 19.5 ± 1.5 km·h^−1^. However, with this low sample size, while the correlations were not significant with the steepest positive slope (i.e., all *r*^2^ <0.44), several correlations were found between the other slopes; i.e., ECR was correlated between level and −5 and +5% slopes (*r*^2^ = 0.66; *p* = 0.050 and *r*^2^ = 0.84; *p* = 0.010, respectively), between −20 and −10% slopes (*r*^2^ = 0.87; *p* = 0.007), between +5 and +10% slopes (*r*^2^ = 0.73; *p* = 0.030), between +15% and −5% and 0% slopes (*r*^2^ = 0.76; *p* = 0.023 and *r*^2^ = 0.79; *p* = 0.018, respectively). Moreover, a tendency was observed between −5 and −10% slopes (*r*^2^ = 0.54; *p* = 0.096), between level and +10% slope (*r*^2^ = 0.58; *p* = 0.078), between +15% and +5% and +10% slopes (*r*^2^ = 0.58; *p* = 0.078 and *r*^2^ = 0.55; *p* = 0.093, respectively).

**Figure 2:**
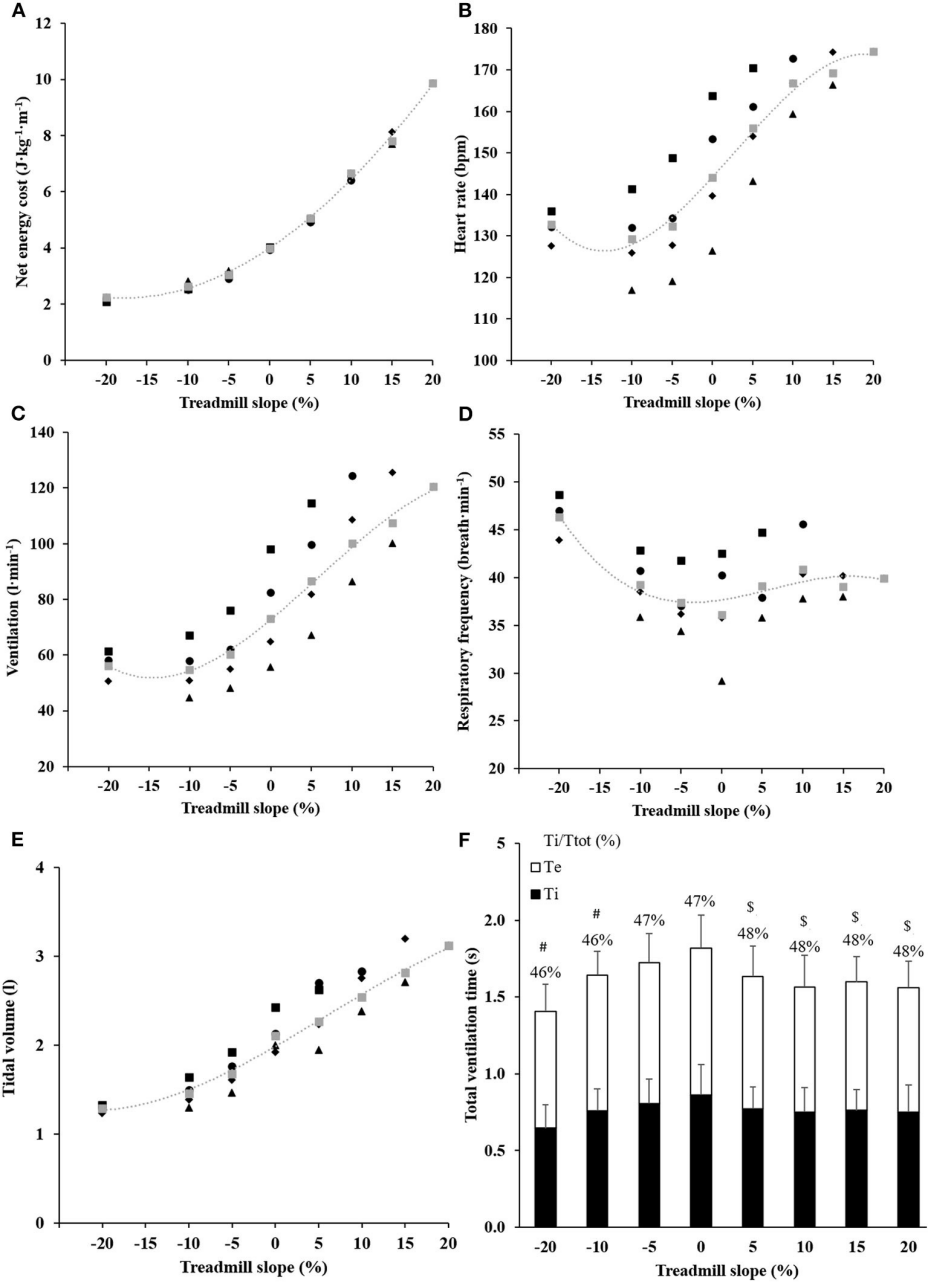
Metabolic and cardiorespiratory responses in inclined running. The energy cost **(A)**, the heart rate **(B)**, the pulmonary ventilation **(C)**, the respiratory frequency **(D)**, and the tidal volume **(E)** of running at different speeds (▴ 8 km·h^−1^, υ 10 km·h^−1^, • 12 km·h^−1^, ■ 14 km·h^−1^, ■ By pooling all speeds) and slopes. For energy cost, fixed effects are calculated by pooling all speeds: slope effect: *p* < 0.001, speed fixed: *p* = 0.006, slope*speed interaction effect: *p* = 0.180. SD has been omitted for clarity. Panel **(F)** shows the ratio of inspiration time (Ti in black) and breathing duty cycle time as a function of treadmill slope. *Error bars* show SD; ^#^*p* < 0.05 vs. 0% slope; ^$^*p* < 0.05 vs. −20% slope.

Values of ECR were different between all slopes (Figure 2A; all *p* < 0.001), between 8 km·h^−1^ and all other speeds at −10% slope and at 8 vs. 10 and 12 km·h^−1^ at −5% slope (all *p* ≤ 0.011). ECR remained similar in between all other speeds at a given slope.

### Optimum Treadmill Slope

The optimum treadmill slope was computed as −17.3 ± 2.3% (Figure 2A), when averaging the optimal slopes across all speeds (Table 2).

**Table 2:** Regression coefficients and optimum treadmill slope for each running speed.

**Speed (km·h^**−1**^)**	***a***	***b***	***c***	***d***	***r*^2^**	**Optimum slope (%)**
8	199.44	8.9189	0.2657	−0.0024	0.998	−14.09^¤^
10	190.56	8.913	0.2686	0.0007	0.998	−17.84
12	187.8	8.9383	0.2639	0.0005	0.996	−17.84
14	192.24	8.9895	0.2283	−0.00009	0.998	−19.46^¤^

*The regression coefficients are for the equation C = a + bi + ci^2^ + di^3^ for each running speed, where C is the oxygen cost of running in ml·kg^−1^·km^−1^ and i is the slope in %*.

¤ *indicate a statistically significant difference vs. 10 and 12 km·h^-1^ (p < 0.05)*.

### Cardiorespiratory Responses at Various Slopes

Heart rate described a sigmoid shape throughout slopes with a plateau in between all negative slopes (all *p* ≥ 0.575, by pooling all speeds; Figure 2B). Heart rate values were correlated with ECR at all slopes (all 0.42 ≤ *r*^2^ ≤ 0.49; *p* ≤ 0.012 by pooling all speeds), except at the steepest positive slope (i.e., +20%).

Pulmonary ventilation, tidal volume, and respiratory frequency did not correlate with ECR, except ventilation at −10% slope and a tendency in −20% (*r*^2^ = 0.16; *p* < 0.032 and *r*^2^ = 0.15; *p* = 0.083, respectively). Pulmonary ventilation remained similar between −10 and −20% slopes (*p* > 0.667 for all speeds; Figure 2C). Tidal volume was lower and respiratory frequency higher at −20% slope than on all other slopes (all *p* ≤ 0.01; Figures 2D,E).

The breathing duty cycle correlated with ECR only at the slopes steeper than ±5%, except for +20% (0.14 ≤ *r*^2^ ≤ 0.42; all *p* ≤ 0.042 and *r*^2^ = 0.06; *p* = 0.642, respectively). The breathing duty cycle was reduced in downhill (≤ −10% slope) vs. level running (both *p* ≤ 0.009; Figure 2F, Table 1).

### Neuromuscular Component at Various Slopes

The mean isometric maximal voluntary KE torque was 247.6 ± 57.6 Nm.

**Figure 3:**
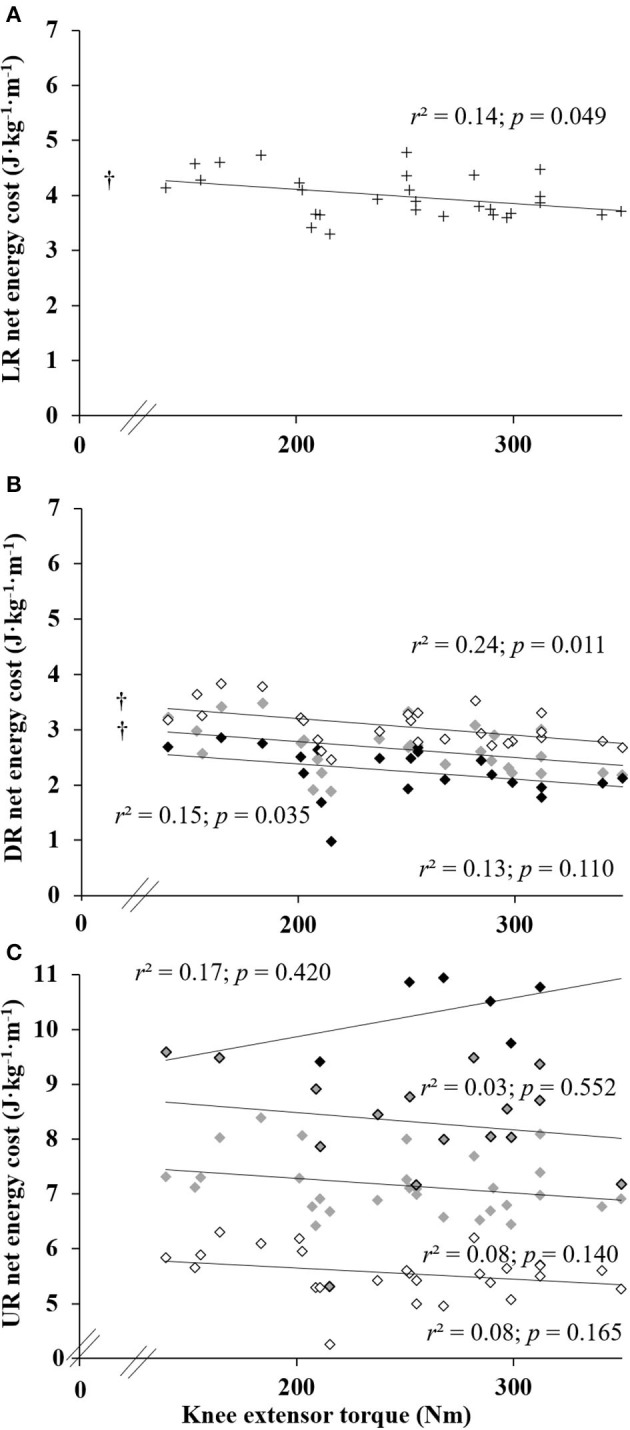
Relationships between the knee extensor torque and the energy cost of running in level (LR) **(A)**, downhill (DR) **(B)** and uphill running (UR) **(C)**. Symbols “+” for 0%, ↓ for ±5%, υ for ±10%, υ for +15% and υ for ±20%; † indicate a statistically significant correlation (*p* < 0.05).

When all running speeds were pooled, ECR was moderately inversely correlated with KE isometric maximal torque at level and negative slopes (Figure 3; 0.14 ≤ *r*^2^ ≤ 0.24; all *p* ≤ 0.049), except at −20% slope. That is, better ECR was associated with greater strength. However, uphill ECR was not correlated with strength.

For a given running speed, the inverse correlations between KE maximal torque and net OCR and ECR mainly on negative slopes were weak (i.e., −10% at 12 and 14 km·h^−1^, −5% at 8 and 10 km·h^−1^, and 0% at 10 km·h^−1^, all *r*^2^ ≥ 0.17; *p* ≤ 0.035) and a similar tendency appeared at several other negative slopes (i.e., −20% at 12 km·h^−1^, −10% at 10 km·h^−1^, and −5% at 12 km·h^−1^, all *r*^2^ ≥ 0.11; *p* ≤ 0.084).

### Biomechanics in Inclined Running

With all running speeds pooled, ECR was neither correlated with ground contact time nor with aerial time. ECR only correlated with step frequency at −20% slope, step length at −10% slope, and maximal ground reaction force at −5% slope (all *r*^2^ ≥ 0.20; *p* ≤ 0.024). The biomechanical duty cycle correlated with ECR in level running at 8 and 10 km·h^−1^ (both *r*^2^ ≥ 0.16; *p* ≤ 0.036) and in the shallow positive slope (i.e., +5% at 8 km·h^−1^, *r*^2^ = 0.24; *p* = 0.03).

## Discussion

This study provides the first comprehensive study of running economy over a wide range of speeds and slopes in recreational athletes. The main findings showed that the ECR values were correlated between all slopes, except between level running and the steepest positive slope, and were correlated with KE torque in level and downhill running. The lowest OCR was estimated between −14% and −20% slopes at speeds close to trail running paces (i.e., between 8 and 14 km·h^−1^), characterized by running economy's correlation with cardiorespiratory (e.g., heart rate, breathing duty cycle, and a tendency for pulmonary ventilation) and step frequency responses.

### Oxygen and Energy Costs in Inclined Running

Oxygen cost and ECR were correlated between all slope conditions for a given running speed, except between level and the steepest uphill slope. These results extend recent findings on the relationships in running economy between −5, 0, and +7.5% slopes at similar $\dot{\text{V}}{\text{O}}_{2}$, where running economy was expressed in mlO_2_·min^−1^·kg^−1^ (Breiner et al., 2018). Specifically, the present study revealed that the intercorrelations diminish at steeper slopes, e.g., +20%. The absence of ECR's correlation between level and the steepest uphill slope partly confirms previous results of Balducci et al. (2016) who found no correlation between level and +12.5 or +25% slopes in maximal incremental uphill running tests, whereas OCR level was correlated with a less steep slope (i.e., +10%) (Balducci et al., 2017). The underpinning mechanisms that may explain the correlation between all low to moderate slopes are two-fold. First, the OCR is almost proportional to the terrain inclination at positive slopes below +15% (Minetti et al., 1994, 2002), according to various mixtures of negative and positive mechanical works, due to elastic storage and release contribution, and their very different metabolic efficiencies (Minetti et al., 1994). Second, this mixture of positive and negative work is associated with the gait stretch-shortening cycle involving the bouncing mechanisms (Dewolf et al., 2016). Meanwhile, the underlying explanation of the absence of ECR's correlation between the level and the steepest uphill slope can be related to the predominant contribution of positive work when considering slopes above +15% (Minetti et al., 1994). The low sample size at the steepest slopes may be seen as a limitation. However, by itself, the substantial decrease in the number of subjects able to run at these slopes (from *N* = 16 to *N* = 6) is an interesting result and confirms the specificity of the highest slopes of our study. Indeed, the efficiency of uphill locomotion at +20% slope tends to become equal to that of concentric muscular work (Margaria et al., 1963); thus, most of the work is to lift the body; the elastic energy storage and recovery (Snyder et al., 2012) and the bouncing mechanisms (Dewolf et al., 2016) are lost. Consequently, the stretch-shortening cycle mechanism disappears, exacerbating the metabolic demand, as assessed by the higher blood lactate values and the cardiorespiratory responses.

### Optimum Treadmill Slope

Negative slopes were characterized by an energetically optimal (i.e., lowest OCR) slope estimated at −17.3%. Although the −15% slope was not tested, to our knowledge, the present study is the most comprehensive one combining several speeds and slopes. The present results extend the existing knowledge (Minetti et al., 1994, 2002), by showing an optimal slope inversely proportional to the running speed (range −14.1 to −19.5% slope; Table 2). Minetti et al. (1994) first calculated an optimum slope close to −10.6% by using the same formula as in this work but with a limited range of slopes and a small sample size (i.e., *N* ≤ 5) in elite sky-runners. The same author later estimated this optimum slope at −20.0% with a much wider range of slopes (Minetti et al., 2002). The fitness level likely changed the overall OCR level, with top athletes allowing a wider range of slopes and showing better economy. Henceforth, the results of this study show that running speed should be increased (Table 2) to improve energy rates at steeper negative slopes, likely to increase the stretch-shortening cycle.

### Cardiorespiratory Responses at Various Slopes

The present results are highlighting downhill's ECR to be correlated with the heart rate, the breathing duty cycle, and a tendency to the pulmonary ventilation. While ventilation increased with increasing positive slope, a “more superficial” tachypneic ventilation pattern appeared (i.e., higher respiratory frequency and lower tidal volume). This result is partly consistent with a previous cardiorespiratory comparison between downhill and uphill running at the lower slope and speed (i.e., ±15% and 8.5 km·h^−1^, respectively), but performed with a respiratory exchange ratio >1.00 in uphill condition (Lemire et al., 2018). Moreover, in the present study, the breathing duty cycle was correlated with ECR at negative slopes and was reduced in all downhill vs. level running at 8 km·h^−1^, but greater in almost all uphill vs. level running at 10 km·h^−1^. Taking these results together, the more superficial ventilation could be due, on the one hand, to the dominant eccentric muscle action in downhill triggering a high heart rate response (Lemire et al., 2020a), and on the other hand, to the limited increases in the end-inspiratory lung volume because of the required trunk stabilization (Lipski et al., 2018), even if further studies are required to investigate the mechanisms underpinning these cardiorespiratory responses.

### Neuromuscular Component Is Implicated in Downhill Running Economy

Oxygen cost and ECR were correlated with KE strength in level and almost all downhill conditions, but not in uphill conditions, showing a role of lower limb muscle strength in ECR, especially when bouncing mechanism is more implicated (Dewolf et al., 2016). The importance of strength training for improving running economy on level [i.e., track (Paavolainen et al., 1999), marathon (Jones, 2006), or triathlon (Millet et al., 2002)] running has been known for a long time, but to our knowledge, this study is the first to report such a correlation between downhill ECR and the KE strength. Downhill running exacerbates lower limb neuromuscular fatigue (Giandolini et al., 2016), and especially KE fatigue (e.g., −15% torque after 15 min at −15% slope) (Lemire et al., 2020b). This high level of muscle activation can induce severe lower limb tissue damage, indirectly evidenced by increases in plasma creatine kinase and myoglobin concentrations or inflammatory markers associated with specific mechanics. Ground reaction forces developed on negative slopes put more strain on the tendons than on the muscles: Muscle contraction would be close to isometric, and the energy would be accumulated in the tendon during a fast stretch and restituted later by a slower muscle lengthening (Roberts and Azizi, 2011). The correlation found between level/downhill ECR and KE strength may be linked with the similar ground reaction forces observed between these conditions (Table 1). The stretch-shortening cycle is meanwhile exacerbated during downhill running. Therefore, it is not surprising that downhill ECR is—at least partly—determined by the strength of the KE. The present study is also suggesting that neuromuscular components, particularly during the eccentric phase (i.e., breaking phase), are paramount for ECR, especially in level and downhill running. Since this eccentric contraction (and the importance of the stretch-shortening cycle) decreases with increasing positive slopes (Snyder et al., 2012), it seems logical that we did not find any relationship between uphill ECR and KE strength.

The absence of correlation between OCR or ECR in uphill running and lower limb muscle strength suggests that KE maximal isometric strength may not be the key factor affecting ECR in uphill running in casual runners. The relationship between lower limb strength and uphill ECR remains debated. For instance, KE isometric torque was positively associated with the performance during a 75-km mountain race, but not with OCR in uphill running (+10% slope) in well-trained runners (Balducci et al., 2017). The maximal lower limb strength was negligible (i.e., 2.8%), compared to velocity at $\dot{\text{V}}{\text{O}}_{2\text{max}}$ accounting for 68.3% of the total regression effect in a 5-km uphill running performance in highly trained runners (Lemire et al., 2021). In the same line, KE maximal isometric strength did not correlate with the uphill running velocity at $\dot{\text{V}}{\text{O}}_{2\text{max}}$ in a homogeneous group of well-trained runners (Lemire et al., 2020a). Overall, the lower limb muscle strength seems negligible for uphill OCR.

### Biomechanics of Inclined Running

Uphill conditions were characterized by higher step frequency and lower step length compared to level, whereas downhill conditions were characterized by shorter ground contact time. Maximum vertical ground reaction force was only correlated with ECR on a moderate negative slope and was higher when combining a substantial negative slope (i.e., −10%) and high running speed vs. level. From a biomechanical standpoint, these results are somewhat consistent with previous studies using a less steep negative slope (i.e., −15%) (Gottschall and Kram, 2005), but different from studies using a moderate negative slope (i.e., −10%) (Vernillo et al., 2020), suggesting a relationship between the maximum vertical ground reaction force and the increasing eccentric muscle action associated with the greater braking forces at the steepest negative slopes (Gottschall and Kram, 2005).

## Conclusion

The present study investigated the physiological, biomechanical, and muscle strength determinants of ECR over a wide range of slopes and speeds and showed a correlation among slopes, except for the steepest positive ones, in healthy recreational runners. Downhill and level ECR appeared to be correlated with KE maximal strength in this group of heterogeneous levels. The present study highlights that on steep slopes (~20%), running energetics are determined by different mechanisms (i.e., reduced bouncing mechanism and muscle higher strength in negative slope vs. higher cardiopulmonary fitness level in positive slope) than on shallow slopes. This has some practical application for mountain and trail runners who experience a large variety of terrains and slopes (Giovanelli et al., 2016; Jeker et al., 2020). These results provide further evidence that inclined running is different than level running, and these unique combined physiological, biomechanical, and neuromuscular strategies may have consequences for training and performance in trail running. An understanding of the factors affecting the downhill running economy at high velocity on steep slopes is of particular interest.

## Data Availability Statement

The raw data supporting the conclusions of this article will be made available by the authors, without undue reservation.

## Ethics Statement

The studies involving human participants were reviewed and approved by commission cantonale d'éthique de la recherche sur l'être humain du canton de vaud. The patients/participants provided their written informed consent to participate in this study. Written informed consent was obtained from the individual(s) for the publication of any potentially identifiable images or data included in this article.

## Author Contributions

GM, FM, and KA conceived and designed research. MF and FM conducted experiments and collected data. ML, MF, and FM analyzed data. ML, GM, and FM wrote the manuscript. All authors read and approved the manuscript.

## Conflict of Interest

The authors declare that the research was conducted in the absence of any commercial or financial relationships that could be construed as a potential conflict of interest.
