# Supplementary material for: Level, Uphill, and Downhill Running Economy Values Are Correlated Except on Steep Slopes
Source: Front Physiol. 2021 Jul 1;12:697315. doi: 10.3389/fphys.2021.697315 (PMC8281813; doi:10.3389/fphys.2021.697315)
Supplement: Supplementary file 1 [file Data_Sheet_1.docx]

**Supplementary Table 1**: Cardiorespiratory and biomechanical parameters at 8 km·h^-1^

| Treadmill slope (%) | -10 | -5 | 0 | +5 | +10 | +15 | +20 |
| --- | --- | --- | --- | --- | --- | --- | --- |
| N | 29 | 26 | 28 | 26 | 29 | 14 | 6 |
| Corrected V̇O_2_ (ml·min^-1^·kg^-1^) | 23.8 ± 3.8 * | 26.2 ± 2.9 * | 31.4 ± 3.2 * | 38.4 ± 3.4 * | 48.3 ± 4.1 * | 54.5 ± 7.1 * | 67.5 ± 4.2 * |
| V̇O_2_ (l·min^-1^) | 1.6 ± 0.3 # | 1.8 ± 0.3 # | 2.2 ± 0.4 | 2.6 ± 0.5 # | 3.2 ± 0.5 # | 3.7 ± 0.6 # | 4.3 ± 0.7 # |
| RER | 0.77 ± 0.05 # | 0.79 ± 0.04 # | 0.84 ± 0.06 | 0.83 ± 0.05 | 0.89 ± 0.05 # | 0.93 ± 0.06 # | 1.00 ± 0.03 # |
| Corrected net energy cost (J·kg^-1^·m^-1^) | 2.8 ± 0.6 * | 3.2 ± 0.4 * | 4.0 ± 0.5 * | 5.1 ± 0.5 * | 6.7 ± 0.6 * | 7.7 ± 1.0 * | 9.9 ± 0.6 * |
| Corrected net metabolic cost (mlO_2_·kg^-1^·km^-1^) | 141.1 ± 28.5 * | 158.7 ± 21.5 * | 198.1 ± 23.9 * | 250.2 ± 25.6 * | 324.8 ± 31.1 * | 371.0 ± 52.9 * | 469.0 ± 31.6 * |
| V̇CO_2_ (l·min^-1^) | 1.2 ± 0.3 # | 1.4 ± 0.2 # | 1.8 ± 0.4 | 2.2 ± 0.4 # | 2.8 ± 0.5 # | 3.4 ± 0.6 # | 4.3 ± 0.7 # |
| V̇_E_ (l·min^-1^) | 44.5 ± 8.7 # | 48.0 ± 7.4 # | 55.6 ± 11.6 * | 67.2 ± 14.0 * | 86.4 ± 17.5 * | 100.0 ± 17.4 * | 120.4 ± 18.9 * |
| RF (breaths·min^-1^) | 36 ± 6 # | 34 ± 6 # | 29 ± 6 | 36 ± 8 # | 38 ± 9 # | 38 ± 8 # | 40 ± 7 # |
| TV (l) | 1.3 ± 0.3 * | 1.5 ± 0.3 * | 2.0 ± 0.5 | 1.9 ± 0.4 # | 2.4 ± 0.5 * | 2.7 ± 0.6 * | 3.1 ± 0.8 * |
| Ti (s) | 0.79 ± 0.13 # | 0.83 ± 0.17 # | 1.04 ± 0.26 | 0.82 ± 0.17 # | 0.80 ± 0.19 # | 0.78 ± 0.15 # | 0.75 ± 0.17 # |
| Te (s) | 0.99 ± 0.21 # | 1.01 ± 0.18 # | 1.16 ± 0.27 | 0.96 ± 0.24 # | 0.89 ± 0.24 # | 0.87 ± 0.20 # | 0.81 ± 0.17 # |
| Ti/Ttot (%) | 45 ± 3 # | 45 ± 3 # | 47 ± 3 | 46 ± 2 | 48 ± 3 | 47 ± 2 | 48 ± 2 |
| HR (bpm) | 117 ± 19 # | 119 ± 16 # | 126 ± 18 * | 143 ± 19 * | 159 ± 20 * | 166 ± 14 * | 174 ± 10 * |
| b[La] (mmol·l^-1^) | - | - | 2.0 ± 0.9 | 2.1 ± 0.9 | 3.9 ± 2.1 # | 4.5 ± 2.8 # | 7.5 ± 1.9 # |
| RPE | 8 ± 1 | 8 ± 1 | 9 ± 2 | 10 ± 2 # | 13 ± 2 # | 14 ± 2 # | 16 ± 2 # |
| Stride length (cm) | 86 ± 4 | 87 ± 5 | 85 ± 3 | 83 ± 3 | 82 ± 4 # | 81 ± 4 # | 82 ± 3 |
| Stride frequency (Hz) | 2.58 ± 0.12 | 2.57 ± 0.14 | 2.61 ± 0.10 | 2.67 ± 0.10 | 2.72 ± 0.13 # | 2.76 ± 0.15 # | 2.71 ± 0.09 |
| Contact time (ms) | 316 ± 30 | 323 ± 31 | 315 ± 40 | 311 ± 20 | 308 ± 41 | 312 ± 23 | 322 ± 12 |
| Fly time (ms) | 72 ± 24 | 67 ± 21 | 69 ± 37 | 64 ± 17 | 60 ± 33 | 52 ± 22 | 48 ± 12 |
| Vertical stiffness (kN·m^-1^) | 18.5 ± 3.4 | 18 ± 3.6 | 19.8 ± 5.1 | 19.6 ± 4.2 | 20.5 ± 4.7 | 20.1 ± 3.9 | 20.6 ± 3.2 |
| Fz (N) | 1249 ± 190 | 1212 ± 178 | 1283 ± 334 | 1216 ± 217 | 1202 ± 228 | 1135 ± 173 | 1194 ± 106 |
| Δy (cm) | 6.8 ± 0.7 | 6.8 ± 0.7 | 6.6 ± 0.6 | 6.3 ± 0.6 | 5.9 ± 0.6 # | 5.8 ± 0.9 # | 5.9 ± 0.6 |

**Supplementary Table 2**: Cardiorespiratory and biomechanical parameters at 10 km·h^-1^

| Treadmill slope (%) | -20 | -10 | -5 | 0 | +5 | +10 | +15 |
| --- | --- | --- | --- | --- | --- | --- | --- |
| N | 20 | 29 | 25 | 26 | 21 | 23 | 6 |
| Corrected V̇O_2_ (ml·min^-1^·kg^-1^) | 23.9 ± 3.6 # | 26.8 ± 4.0 # | 29.8 ± 2.9 # $ | 37.2 ± 3.0 $ | 44.6 ± 4.7 # $ | 57.0 ± 3.5 # $ | 69.3 ± 4.6 # $ |
| V̇O_2_ (l·min^-1^) | 1.7 ± 0.3 # | 1.8 ± 0.4 # | 2.0 ± 0.3 # $ | 2.5 ± 0.4 $ | 3.0 ± 0.5 # $ | 3.8 ± 0.6 # $ | 4.5 ± 0.4 # $ |
| RER | 0.75 ± 0.04 # | 0.77 ± 0.04 # | 0.81 ± 0.05 $ | 0.83 ± 0.06 $ | 0.90 ± 0.08 # $ | 0.97 ± 0.07 # $ | 1.00 ± 0.02 # $ |
| Corrected net energy cost (J·kg^-1^·m^-1^) | 2.3 ± 0.4 * | 2.6 ± 0.5 * | 3.0 ± 0.3 * | 3.9 ± 0.4 * | 4.9 ± 0.6 * | 6.5 ± 0.5 * | 8.1 ± 0.6 * |
| Corrected net metabolic cost (mlO_2_·kg^-1^·km^-1^) | 113.4 ± 21.6 * | 130.6 ± 23.7 * | 148.5 ± 17.4 * | 193.5 ± 18.1 * | 237.4 ± 27.9 * | 312.1 ± 21.1 * | 385.5 ± 27.4 * |
| V̇CO_2_ (l·min^-1^) | 1.3 ± 0.2 # | 1.4 ± 0.3 # | 1.6 ± 0.3 # $ | 2.1 ± 0.4 $ | 2.7 ± 0.4 # $ | 3.7 ± 0.7 # $ | 4.5 ± 0.4 # $ |
| V̇_E_ (l·min^-1^) | 50.6 ± 9.3 # | 50.9 ± 9.8 # | 54.9 ± 10.1 # | 64.9 ± 11.5 $ | 81.8 ± 13.4 # $ | 108.5 ± 23.9 # $ | 125.4 ± 20.6 # $ |
| RF (breaths·min^-1^) | 44 ± 9 # | 39 ± 7 $ | 36 ± 8 $ | 36 ± 9 $ | 38 ± 8 $ | 40 ± 8 # | 40 ± 8 # |
| TV (l) | 1.2 ± 0.3 # | 1.4 ± 0.3 # | 1.6 ± 0.5 # $ | 1.9 ± 0.5 $ | 2.2 ± 0.5 # $ | 2.8 ± 0.6 # $ | 3.2 ± 0.6 # $ |
| Ti (s) | 0.68 ± 0.17 # | 0.78 ± 0.17 | 0.83 ± 0.20 $ | 0.84 ± 0.21 $ | 0.79 ± 0.13 $ | 0.75 ± 0.16 # | 0.75 ± 0.15 # |
| Te (s) | 0.81 ± 0.20 # | 0.90 ± 0.17 | 0.96 ± 0.24 $ | 0.98 ± 0.27 $ | 0.86 ± 0.18 | 0.82 ± 0.24 # | 0.80 ± 0.18 # |
| Ti/Ttot (%) | 46 ± 3 | 46 ± 4 | 47 ± 3 | 47 ± 3 | 48 ± 3 $ | 48 ± 3 $ | 48 ± 2 |
| HR (bpm) | 128 ± 16 # | 126 ± 21 # | 128 ± 16 # | 140 ± 18 $ | 154 ± 18 # $ | 167 ± 15 # $ | 174 ± 10 # $ |
| b[La] (mmol·l^-1^) | - | - | - | 2.3 ± 0.8 * | 3.6 ± 2.2 * | 5.4 ± 2.4 * | 7.2 ± 3.5 * |
| RPE | 9 ± 2 | 9 ± 2 | 9 ± 2 | 10 ± 2 | 12 ± 2 # $ | 15 ± 2 # $ | 15 ± 2 # $ |
| Stride length (cm) | 1.04 ± 0.04 | 1.04 ± 0.04 | 1.04 ± 0.05 | 1.03 ± 0.04 | 1.01 ± 0.05 | 0.99 ± 0.04 # $ | 0.99 ± 0.04 # $ |
| Stride frequency (Hz) | 2.69 ± 0.10 | 2.67 ± 0.10 | 2.67 ± 0.12 | 2.70 ± 0.11 | 2.75 ± 0.14 | 2.82 ± 0.12 # $ | 2.81 ± 0.11 # $ |
| Contact time (ms) | 279 ± 32 | 282 ± 30 | 290 ± 29 $ | 289 ± 25 | 288 ± 28 $ | 289 ± 25 $ | 283 ± 16 |
| Fly time (ms) | 94 ± 32 # | 93 ± 27 # | 85 ± 23 | 82 ± 21 $ | 77 ± 20 $ | 66 ± 17 # $ | 74 ± 16 # $ |
| Vertical stiffness (kN·m^-1^) | 21.7 ± 4.2 | 21.0 ± 3.4 | 20.4 ± 3.7 | 21.2 ± 4.0 | 21.3 ± 3.9 | 22.6 ± 3.8 # | 23.2 ± 3.5 # |
| Fz (N) | 1412 ± 230 | 1393 ± 209 # | 1332 ± 188 | 1357 ± 231 | 1306 ± 226 $ | 1284 ± 179 # $ | 1353 ± 114 $ |
| Δy (cm) | 6.6 ± 0.7 | 6.7 ± 0.6 | 6.6 ± 0.6 | 6.4 ± 0.6 | 6.2 ± 0.6 | 5.7 ± 0.5 # $ | 5.9 ± 0.6 # $ |

**Supplementary Table 3**: Cardiorespiratory and biomechanical parameters at 12 km·h^-1^

| Treadmill slope (%) | -20 | -10 | -5 | 0 | +5 | +10 |
| --- | --- | --- | --- | --- | --- | --- |
| N | 19 | 28 | 21 | 29 | 16 | 10 |
| Corrected V̇O_2_ (ml·min^-1^·kg^-1^) | 27.1 ± 4.9 # | 30.2 ± 3.9 # | 34.0 ± 4.8 # $ | 43.6 ± 5.2 $ | 52.2 ± 5.9 # $ | 65.9 ± 2.5 # $ |
| V̇O_2_ (l·min^-1^) | 1.8 ± 0.3 # | 2.1 ± 0.4 # | 2.3 ± 0.4 # $ | 3.0 ± 0.5 $ | 3.6 ± 0.6 # $ | 4.2 ± 0.6 # $ |
| RER | 0.77 ± 0.08 # | 0.79 ± 0.04 # | 0.80 ± 0.05 | 0.86 ± 0.08 # $ | 0.97 ± 0.10 # $ | 1 ± 0.05 # $ |
| Corrected net energy cost (J·kg^-1^·m^-1^) | 2.2 ± 0.5 # | 2.5 ± 0.4 # | 2.9 ± 0.5 # $ | 3.9 ± 0.6 $ | 4.9 ± 0.6 # $ | 6.4 ± 0.3 # $ |
| Corrected net metabolic cost (mlO_2_·kg^-1^·km^-1^) | 110.7 ± 24.7 # | 125.9 ± 19.6 # | 145.2 ± 23.9 # $ | 293.1 ± 26.0 $ | 236.2 ± 29.6 # $ | 304.7 ± 12.7 # $ |
| V̇CO_2_ (l·min^-1^) | 1.4 ± 0.3 # | 1.6 ± 0.3 # | 1.9 ± 0.3 # | 2.5 ± 0.5 $ | 3.4 ± 0.7 # $ | 4.2 ± 0.6 # $ |
| V̇_E_ (l·min^-1^) | 58.2 ± 11.6 # | 57.8 ± 10.7 # | 62.1 ± 9.7 # | 82.4 ± 16 $ | 99.7 ± 19 # $ | 124.3 ± 21.9 # $ |
| RF (breaths·min^-1^) | 47 ± 9 # | 41 ± 9 | 37 ± 8 $ | 40 ± 9 $ | 38 ± 7 $ | 46 ± 12 |
| TV (l) | 1.3 ± 0.3 # | 1.5 ± 0.3 # | 1.8 ± 0.4 # $ | 2.1 ± 0.5 $ | 2.7 ± 0.6 # $ | 2.8 ± 0.6 # $ |
| Ti (s) | 0.63 ± 0.14 | 0.73 ± 0.16 | 0.83 ± 0.21 $ | 0.75 ± 0.17 | 0.79 ± 0.16 $ | 0.68 ± 0.17 |
| Te (s) | 0.75 ± 0.17 | 0.84 ± 0.16 | 0.91 ± 0.23 $ | 0.83 ± 0.18 | 0.86 ± 0.18 | 0.73 ± 0.23 |
| Ti/Ttot (%) | 46 ± 3 | 47 ± 3 | 48 ± 3 | 48 ± 3 | 48 ± 2 | 48 ± 3 |
| HR (bpm) | 132 ± 18 # | 132 ± 21 # | 134 ± 18 # | 153 ± 20 $ | 161 ± 14 $ | 173 ± 11 # $ |
| b[La] (mmol·l^-1^) | - | - | 3.3 ± 1.6 | 3.5 ± 1.4 | 4.5 ± 2.8 | 6.4 ± 3.1 # |
| RPE | 10 ± 2 # | 10 ± 2 # | 9 ± 2 # | 11 ± 2 $ | 13 ± 2 # $ | 16 ± 3 # $ |
| Stride length (cm) | 1.19 ± 0.04 | 1.22 ± 0.05 | 1.21 ± 0.06 | 1.19 ± 0.05 | 1.17 ± 0.07 | 1.12 ± 0.05 |
| Stride frequency (Hz) | 2.80 ± 0.10 | 2.74 ± 0.11 | 2.76 ± 0.13 | 2.82 ± 0.11 | 2.85 ± 0.16 | 2.97 ± 0.13 |
| Contact time (ms) | 248 ± 23 | 255 ± 25 | 257 ± 27 | 262 ± 25 | 261 ± 25 | 251 ± 17 |
| Fly time (ms) | 109 ± 29 | 110 ± 24 # | 106 ± 22 | 94 ± 21 | 91 ± 20 | 86 ± 17 |
| Vertical stiffness (kN·m^-1^) | 23.7 ± 3.8 | 23.7 ± 3.3 | 22.9 ± 4.3 | 23.6 ± 3.9 | 23.4 ± 4.5 | 25.2 ± 2.4 |
| Fz (N) | 1459 ± 212 | 1535 ± 229 # | 1455 ± 249 | 1433 ± 244 | 1378 ± 241 | 1371 ± 179 |
| Δy (cm) | 6.2 ± 0.6 | 6.5 ± 0.5 # | 6.4 ± 0.6 | 6.1 ± 0.5 | 6 ± 0.7 | 5.4 ± 0.5 |

**Supplementary Table 4**: Cardiorespiratory and biomechanical parameters at 14 km·h^-1^

| Treadmill slope (%) | -20 | -10 | -5 | 0 | +5 |
| --- | --- | --- | --- | --- | --- |
| N | 11 | 27 | 25 | 20 | 15 |
| Corrected V̇O_2_ (ml·min^-1^·kg^-1^) | 29.3 ± 6.3 * | 34.6 ± 4.1 * | 39.9 ± 3.9 * | 50.5 ± 3.2 * | 61.5 ± 4.6 * |
| V̇O_2_ (l·min^-1^) | 2.1 ± 0.4 # | 2.4 ± 0.4 # | 2.7 ± 0.5 # $ | 3.4 ± 0.6 $ | 3.9 ± 0.7 # $ |
| RER | 0.76 ± 0.06 # | 0.80 ± 0.05 # | 0.85 ± 0.08 # $ | 0.92 ± 0.08 $ | 0.95 ± 0.10 $ |
| Corrected net energy cost (J·kg^-1^·m^-1^) | 2.1 ± 0.5 * | 2.5 ± 0.4 * | 3.0 ± 0.3 * | 4.0 ± 0.3 * | 5.0 ± 0.4 * |
| Corrected net metabolic cost (mlO_2_·kg^-1^·km^-1^) | 104.3 ± 26.9 * | 127.1 ± 17.4 * | 149.4 ± 16.6 * | 194.9 ± 13.5 * | 242.2 ± 19.9 * |
| V̇CO_2_ (l·min^-1^) | 1.6 ± 0.3 # | 1.9 ± 0.3 # | 2.3 ± 0.5 # $ | 3.1 ± 0.5 $ | 3.7 ± 0.5 # $ |
| V̇_E_ (l·min^-1^) | 61.4 ± 11.9 # | 67.1 ± 13 # | 76.1 ± 14 # $ | 98 ± 17.4 $ | 114.5 ± 23.9 # $ |
| RF (breaths·min^-1^) | 49 ± 10 # | 43 ± 9 $ | 42 ± 10 $ | 42 ± 10 $ | 45 ± 10 |
| TV (l) | 1.3 ± 0.3 # | 1.6 ± 0.4 # $ | 1.9 ± 0.5 # $ | 2.4 ± 0.6 $ | 2.6 ± 0.5 $ |
| Ti (s) | 0.60 ± 0.14 # | 0.71 ± 0.18 $ | 0.74 ± 0.18 $ | 0.73 ± 0.21 $ | 0.68 ± 0.16 # |
| Te (s) | 0.73 ± 0.17 | 0.80 ± 0.18 $ | 0.8 ± 0.20 $ | 0.78 ± 0.21 | 0.74 ± 0.19 |
| Ti/Ttot (%) | 46 ± 2 | 47 ± 3 | 48 ± 3 | 48 ± 2 $ | 48 ± 3 $ |
| HR (bpm) | 136 ± 17 # | 141 ± 22 # | 149 ± 19 # | 164 ± 17 $ | 170 ± 12 # $ |
| b[La] (mmol·l^-1^) | - | - | 3.0 ± 1.5 | 4.0 ± 1.8 | 6.5 ± 3.3 # |
| RPE | 11 ± 2 | 10 ± 2 # | 10 ± 3 | 13 ± 2 # | 15 ± 2 # $ |
| Stride length (cm) | 1.36 ± 0.07 | 1.37 ± 0.05 | 1.37 ± 0.06 | 1.36 ± 0.06 | 1.31 ± 0.07 # |
| Stride frequency (Hz) | 2.86 ± 0.15 | 2.84 ± 0.09 | 2.84 ± 0.12 | 2.87 ± 0.12 | 2.98 ± 0.15 # |
| Contact time (ms) | 221 ± 22 # | 231 ± 19 # | 233 ± 19 | 240 ± 27 $ | 232 ± 17 $ |
| Fly time (ms) | 129 ± 32 # | 122 ± 19 # | 120 ± 19 # | 110 ± 26 $ | 105 ± 16 # $ |
| Vertical stiffness (kN·m^-1^) | 26.5 ± 4.9 # | 26.6 ± 3.4 $ | 25.9 ± 4 $ | 26.3 ± 4.6 $ | 28.4 ± 3.8 |
| Fz (N) | 1577 ± 287 # | 1633 ± 234 # | 1584 ± 240 # | 1562 ± 286 $ | 1567 ± 188 # $ |
| Δy (cm) | 6.0 ± 0.7 | 6.1 ± 0.4 # | 6.1 ± 0.5 | 6.0 ± 0.5 | 5.6 ± 0.6 # |

Note. Values are means ± SD, corrected oxygen uptake (corrected V̇O_2_), minute pulmonary ventilation (V̇_E_), respiratory frequency (RF), heart rate (HR), blood lactate concentration (b[La]), rate of perceived exertion (RPE), maximal vertical ground reaction force (Fz), vertical displacement of the center of mass during ground contact (Δy). *Asterisks* (*) indicate a statistically significant difference vs. all other conditions, ^#^ vs. 0% slope and ^$^ vs. -20% slope (*p* < 0.05).

**Supplementary Table 5**: Individual oxygen uptake value correction

| Treadmill slope (%) |  | **-5** | | | |  | **0** | | | |  | **+5** | | | |  | **+10** | | |  | **+15** | |  | **+20** |
| --- | --- | --- | --- | --- | --- | --- | --- | --- | --- | --- | --- | --- | --- | --- | --- | --- | --- | --- | --- | --- | --- | --- | --- | --- |
| Treadmill velocity (km·h^-1^) |  | **8** | **10** | **12** | **14** |  | **8** | **10** | **12** | **14** |  | **8** | **10** | **12** | **14** |  | **8** | **10** | **12** |  | **8** | **10** |  | **8** |
| 1 |  | - | - | - | - |  | - | - | - | 2.1 |  | - | - | 2.4 | - |  | - | 3.8 | - |  | - | - |  | - |
| 2 |  | - | - | - | - |  | - | - | - | 3.5 |  | - | 6.2 | 6.6 | - |  | 1.9 | 4.6 | - |  | - | - |  | - |
| 3 |  | - | - | - | - |  | - | - | - | - |  | - | - | - | 5.0 |  | - | - | 6.5 |  | - | - |  | - |
| 4 |  | - | - | - | - |  | - | - | - | - |  | - | - | 2.4 | - |  | 2.9 | 3.2 | 5.9 |  | 2.8 | - |  | - |
| 5 |  | - | - | - | - |  | - | - | - | - |  | - | - | - | - |  | - | - | - |  | - | - |  | - |
| 6 |  | - | - | - | - |  | - | - | - | - |  | - | - | - | 3.9 |  | - | - | 5.3 |  | - | 6.8 |  | 4.4 |
| 7 |  | - | - | - | 2.5 |  | - | - | 5.1 | - |  | - | 5.6 | - | - |  | 3.6 | - | - |  | - | - |  | - |
| 8 |  | - | - | - | - |  | - | - | - | - |  | - | - | - | 2.6 |  | - | - | - |  | - | 2.6 |  | 4.0 |
| 9 |  | - | - | - | - |  | - | - | - | - |  | - | 1.9 | 6.2 | - |  | 1.8 | 6.5 | - |  | - | - |  | - |
| 10 |  | - | - | - | - |  | - | - | - | 2.9 |  | - | - | - | - |  | 2.9 | 5.4 | - |  | - | - |  | - |
| 11 |  | - | - | - | - |  | - | - | - | - |  | - | - | - | 2.1 |  | - | - | - |  | - | 4.7 |  | 3.4 |
| 12 |  | - | - | - | - |  | - | - | - | 2.3 |  | - | - | - | 9.5 |  | 5.0 | 4.0 | - |  | - | - |  | - |
| 13 |  | - | - | - | - |  | - | - | - | 3.4 |  | - | - | 3.5 | 6.4 |  | - | 4.3 | - |  | 3.4 | - |  | - |
| 14 |  | - | - | - | 2.6 |  | - | - | 3.5 | 5.5 |  | - | 3.5 | - | - |  | 5.1 | - | - |  | - | - |  | - |
| 15 |  | - | - | - | - |  | - | - | - | - |  | - | - | - | - |  | - | - | 2.2 |  | - | 3.3 |  | - |
| 16 |  | - | - | - | - |  | - | - | - | - |  | - | - | - | - |  | - | 2.9 | - |  | 4.0 | - |  | - |
| 17 |  | - | - | - | - |  | - | - | - | - |  | - | - | - | - |  | - | - | - |  | - | - |  | 6.6 |
| 18 |  | - | - | - | - |  | - | - | - | 3.8 |  | 2.1 | 2.6 | - | - |  | 3.5 | - | - |  | - | - |  | - |
| 19 |  | - | - | - | - |  | - | - | - | 3.5 |  | - | - | 5.1 | - |  | - | 5.0 | - |  | 7.8 | - |  | - |
| 20 |  | - | - | - | - |  | - | - | 2.6 | - |  | - | - | - | - |  | 3.8 | - | - |  | - | - |  | - |
| 21 |  | - | - | - | - |  | - | - | - | - |  | - | - | - | 4.5 |  | - | 1.3 | - |  | 0.9 | - |  | 6.2 |
| 22 |  | - | - | - | - |  | - | - | - | - |  | - | - | - | 0.4 |  | - | - | 6.0 |  | - | - |  | - |
| 23 |  | - | - | 2.5 | - |  | - | - | 3.7 | - |  | - | 3.9 | - | - |  | 5.1 | - | - |  | - | - |  | - |
| 24 |  | - | - | - | - |  | - | - | - | - |  | - | - | - | 2.6 |  | - | - | 4.1 |  | - | - |  | 4.8 |
| 25 |  | - | - | - | 2.1 |  | - | - | 2.6 | - |  | - | 2.6 | - | - |  | 3.2 | - | - |  | - | - |  | - |
| 26 |  | - | - | - | - |  | - | - | - | - |  | - | - | - | - |  | - | 3.6 | - |  | - | - |  | - |
| 27 |  | - | - | - | - |  | - | - | - | - |  | - | - | - | 4.1 |  | - | 2.0 | - |  | - | 8.8 |  | - |
| 28 |  | - | - | - | - |  | - | - | - | 2.1 |  | - | - | 3.2 | - |  | - | 5.2 | - |  | - | - |  | - |
| 29 |  | - | - | - | - |  | - | - | - | 3.4 |  | - | - | 3.5 | 6.4 |  | - | 4.3 | - |  | 4.0 | - |  | - |

Note. Each line represents the oxygen uptake correction for each subject expressed in ml·min^-1^·kg^-1^, based on respiratory exchange ratio and blood lactate values.

**Supplementary Table 6**: The energy cost of running (ECR) per second, based on the theorical ECR (Minetti et al., 2002), and the repartition between the four sessions (S1 to S4).

| **Treadmill Slope (%)** | **Theorical ECR** | **Treadmill Speed (km·h^-1^)** | | | |
| --- | --- | --- | --- | --- | --- |
|  | **(J·kg^-1^·m^-1^)** | **8** | **10** | **12** | **14** |
| **20** | 8.92 | 19.82 |  |  |  |
|  |  | S4 |  |  |  |
| **15** | 7.35 | 16.33 | 20.42 |  |  |
|  |  | S4 | S3 |  |  |
| **10** | 5.77 | 12.82 | 16.03 | 19.23 |  |
|  |  | S2 | S2 | S2 |  |
| **5** | 4.59 | 10.20 | 12.75 | 15.30 | 17.85 |
|  |  | S4 | S3 | S4 | S3 |
| **0** | 3.4 | 7.56 | 9.44 | 11.33 | 13.22 |
|  |  | S1 | S4 | S3 | S2 |
| **-5** | 2.67 | 5.93 | 7.42 | 8.90 | 10.38 |
|  |  | S4 | S3 | S4 | S3 |
| **-10** | 1.93 | 4.29 | 5.36 | 6.43 | 7.51 |
|  |  | S2 | S2 | S2 | S2 |
| **-20** | 1.73 |  | 4.81 | 5.77 | 6.73 |
|  |  |  | S3 | S3 | S4 |

Note. The 8 km·h^-1^ level running condition is performed in the first session, and the other conditions are distributed over the three other sessions to obtain a similar effort on each session. The conditions are then randomized within each session.
